# Porous titanium fiber mesh with tailored elasticity and its effect on stromal cells

**DOI:** 10.1002/jbm.b.34556

**Published:** 2020-01-14

**Authors:** Evy Aerts, Jinmeng Li, Mies J. Van Steenbergen, Tanika Degrande, John A. Jansen, X. Frank Walboomers

**Affiliations:** ^1^ Biomaterials, Department of Dentistry, Radboud Institute for Molecular Life Sciences Radboud University Medical Center Nijmegen The Netherlands; ^2^ Department of Pharmaceutics, Utrecht Institute for Pharmaceutical Sciences Utrecht University Utrecht The Netherlands; ^3^ Bekaert Fibre Technologies Zwevegem Belgium

**Keywords:** bone regeneration, bone‐implant interface, marrow stromal cells, tissue engineering, titanium fiber mesh

## Abstract

Porous titanium fiber mesh (TFM) is considered a suitable scaffold material for bone reconstruction. Also, TFM can be used to cover the surface of bone‐anchored devices, that is, orthopedic or dental implants. The titanium fiber size has an effect of the stiffness as well as porosity of the titanium mesh, which can influence the behavior of bone forming cells. Therefore, the aim of this study was to vary TFM composition, in order to achieve different stiffness, and to assess the effects of such variation on the behavior of bone marrow‐derived stromal cells (BMSCs). With that purpose, nine types of TFM (porosities 60–87%; fiber size 22–50 μm), were examined for their mechanical properties as well as their effect on the proliferation and differentiation of rat bone marrow‐derived stromal cells (rBMSCs) up to 21 days. Dynamic mechanical analysis revealed that the stiffness of TFM were lower than of solid titanium and decreased with larger fiber sizes. The stiffness could effectively be tailored by altering fiber properties, which altered the pore simultaneously. For the 22 and 35 μm size fiber meshes with the highest porosity, the stiffness closely matched the value found in literature for cortical bone. Finally, all tested TFM types supported the growth and differentiation of rBMSCs. We concluded that TFM material has been proven cytocompatible. Further preclinical studies are needed to assess which TFM type is most suitable as clinical use for bone ingrowth and bone regeneration.

## 1 INTRODUCTION

Titanium fiber mesh (TFM) is composed of sintered nonwoven microscale fibers of commercially pure titanium (cpTi) (Jansen, von Recum, van der Waerden, & de Groot, 1992). Due to its porous structure and the biocompatibility of titanium, TFM is considered as a suitable scaffold material for bone reconstruction, when used in combination with bone forming cells and/or signaling molecules (Holtorf, Jansen, & Mikos, 2005). In vitro, these fiber mesh scaffolds were reported to support the adhesion and osteogenic differentiation of primary marrow stromal cells in both static and flow perfusion culture conditions (Bancroft et al., 2002; van den Dolder, Bancroft, et al., 2003a; van den Dolder, Spauwen, & Jansen, 2003b). Also in vivo, mesh loaded with osteoblast precursor cells was shown to enhance bone formation in both orthotopic (Vandendolder, Farber, Spauwen, & Jansen, 2003) as well as ectopic (Vehof, Spauwen, & Jansen, 2000) locations. Further, it was postulated to apply TFM as an outer layer on bone‐anchored devices, that is, orthopedic or dental implants. The application of a porous material is supposed to enhance the mechanical interlocking between the device and bone by bone ingrowth into the mesh porosity (Karageorgiou & Kaplan, 2005; Li et al., 2007) resulting in an increased “osseointegration” compared to a nonporous implant material (Bobyn, Stackpool, Hacking, Tanzer, & Krygier, 1999). Furthermore, the porous structure will support the interfacial stress transfer evoked by the implant loading, and thus may aid in maintaining an enhanced tissue‐to‐implant connection over a prolonged period of time (Henkel et al., 2013).

The above‐mentioned is based on the observation that cells are capable of sensing and responding to mechanical forces, with the help of intercellular signals transduced by mechanical properties of their surrounding extracellular microenvironment, which controls stem cell lineage specification (Discher, Janmey, & Wang, 2005; Engler, Sen, Sweeney, & Discher, 2006). And vice versa, the mechanical properties of materials, that is, stiffness, can also have an effect on the behavior of cells, (Huebsch et al., 2010) such as cell growth (Engler et al., 2006), migration (Janson & Putnam, 2015), and differentiation (Wang & Chen, 2013). As there are many different processes that occur during bone regeneration, it is difficult to build a general criterion for a suitable scaffold stiffness that optimally stimulates this process. Nevertheless, to promote osteoblast precursor differentiation, current knowledge suggests that the scaffold stiffness should match the in vivo stiffness of the skeletal tissue under consideration. The Young's modulus of cortical bone and cancellous bone is 7–30 GPa and 0.05–0.5 GPa, respectively (Henkel et al., 2013). The TFM material, as used in our previous studies, had a fiber size of 50 μm and stiffness of 5 GPa. Currently, thinner titanium fibers (~22 and ~35 μm) can be manufactured. Using stainless steel fibers, it was proven that a decrease in fiber size could has a very drastic effect on the stiffness of a fiber mesh (Jansen et al., 1992). However, a change in fiber size can also has an effect on the fiber mesh porosity resulting in smaller distance between fiber (Paquay, DeBlieckHogervorst, & Jansen, 1996). Therefore, the objective of this study was to vary TFM composition, in order to achieve different stiffness close to cortical bone (7–30 GPa), and to assess the effects of such variation on the proliferation and differentiation of bone marrow‐derived stromal cells (BMSCs). We hypothesized that fiber size and porosity influence the stiffness of TFM, and also that such variation will affect BMSC behavior.

## 2 MATERIALS AND METHODS

### 2.1 TFM characterization

Nine types of TFM sheets were fabricated by and obtained from Bekaert B.V. (Zwevegem, Belgium). The meshes were fabricated by compression and then sintered randomly to bond the fibers at their points of contact. The fibers had a thin rectangular cross‐section, like a ribbon or linguini. TFM types varied in fiber size (22, 35, or 50 μm) and volumetric porosity (60, 70, 75, 80, or 87%) were named accordingly, for example, 22–60 represents the TFM type with a 22 μm fiber size and porosity of 60% (Table 1). To prevent misunderstanding, the fiber size present throughout the manuscript means the length of the longest side of the rectangular cross‐section.

**Table 1:** Abbreviations and characteristics of the various TFM types

TFM type	Sheet thickness (mm)	Fiber diameter (mm)	Volumetric porosity (%)
22–60	0.3	22	60
22–70	0.3	22	70
22–80	0.3	22	80
35–60	0.3	35	60
35–70	0.3	35	70
35–80	0.3	35	80
50–70	0.5	50	70
50–75	0.5	50	75
50–87	1	50	87

Abbreviation: TFM, titanium fiber mesh.

#### 2.1.1 *TFM surface imaging and validation of titanium purity*

TFM structure and morphology were validated with scanning electron microscopy (SEM, Sigma 300, Breda, the Netherlands). The surface of the mesh sheets was imaged at ×250 magnification to obtain an overview of TFM architecture. Energy‐Dispersive X‐ray Spectroscopy (EDS, Quantax EDS for SEM, Bruker Elemental GmbH, Kalkar, Germany) was used to assess the composition of the titanium. Using the same settings as described above, a region of superficial fibers was selected and the atomic composition was determined.

#### 2.1.2 *Analysis of TFM porosity and pore size*

Cross‐sections of TFM were made to examine the distribution of fibers and to assess the total porosity of the mesh. The distance between the fibers, which refers to mesh pore size throughout the manuscript, was also determined via cross‐sections. TFM samples were embedded in methylmethacrylate (MMA) and after polymerization sectioned perpendicularly to the sheet surface using a modified sawing microtome technique (van der Lubbe, Klein, & de Groot, 1988). Samples coated with chromium were visualized with SEM as described above. Using ImageJ (1.48v for Microsoft, National Institutes of Health, MD), a region of interest (ROI) was drawn within the sample and a global color threshold was used to distinguish between fibers and surrounding MMA. Total porosity was calculated as the percentage of MMA within the ROI. Subsequently, to determine the mesh pore size, 15 rods (cut cross‐sections of the fibers in the MMA matrix) from the ROI of each cross‐section sample were selected and the distance between the rod and the ambient fibers were measured by ImageJ. The mean and *SD* of these measurements was calculated and considered as the mesh pore size.

Additionally, theoretical porosity was computed based on the TFM and bulk titanium densities. Area density (g/m^2^) of all sheets was determined and multiplied by sheet thickness to obtain the density of TFM (*ρ*TFM), which was divided by the density of solid titanium (*ρ*Ti = 4.51 × 10^3^ kg/m^3^) to calculate the theoretical porosity of the TFM.

$$\text{Theoretical porosity}\;\left(\%\right)=\left(1-\frac{\rho \mathrm{TFM}}{\rho \mathrm{Ti}}\right)\times 100\%$$

The theoretical porosity of TFM is the percentage void space calculated by matrix density, whereas the total porosity determined here is the fraction of the surface area between fibers from the cross‐section.

#### 2.1.3 *Dynamic mechanical analysis*

Mechanical properties of the meshes were tested with dynamic mechanical analysis (DMA). In triplicate, strips of 6 × 1.3 cm were cut from TFM sheets and analyzed with DMA Q800 (TA Instruments, New Castle, DW). Using dual cantilever measurements, the strips were subjected to a controlled, cyclic stress/strain, which deforms the mesh to a certain amount. The deformation is related to the stiffness of the mesh material. Frequency and strain sweeps were first performed to optimize conditions. All reported measurements were thereafter performed at room temperature (RT) with a frequency of 1 Hz and an amplitude of 1 μm. The stiffness was calculated from stress, strain and phase lag using TA Software (Advantage™ Software for Windows, Universal Analysis, v5.5.22).

### 2.2 TFM scaffold production

Scaffolds with a diameter of 4 mm were punched out from TFM sheets using a metal socket punch, cleaned ultrasonically in isopropanol for 10 min and dried to air. Prior to cell culture experiments, scaffolds were sterilized by autoclavation at 121°C, transferred to 24‐wells plates, and treated with radiofrequency glow discharge treatment for 5 min (RFGD; Plasma cleaner/sterilizer, Harrick Scientific, Pleasantville, NJ) to increase wettability.

### 2.3 Cell viability

Mouse osteoblast precursor cells MC3T3‐E1 (ATCC®, LGC Standards GmbH, Wesel, Germany) were seeded onto TFM scaffolds to determine cell viability. Cells were cultured in complete medium (α‐minimum essential medium [α‐MEM, Gibco®, Life Technologies B.V., Breda, the Netherlands] supplemented with 10% Fetal Bovine Serum [FBS, Sigma‐Aldrich Chemie GmbH, Taufkirchen, Germany]) at 37°C and 5% CO_2_, and were transferred twice a week at 80–90% confluency. Prior to seeding, cells were enzymatically dissociated and re‐suspended in complete medium diluted to 200,000 cells/ml.

Four scaffolds of each TFM type were transferred to one well of a 24‐wells suspension plate (Costar® 24 well plate, Corning Incorporated, Kennebunk, ME) containing 200,000 MC3T3 cells in 1 ml complete medium (50,000 cells/scaffold). This plate was incubated at 37°C and 5% CO_2_ for 3 hr on a rotating device at 2 Hz while tilted at an angle of 50° (dynamic seeding). Two scaffolds of each type were imaged immediately, while two others were cultured up to 24 hr before imaging.

Prior to imaging, the scaffolds were gently washed in PBS and placed on glass microscopy slides. A Live/Dead assay was performed according to the manufacturer's protocol (Live/Dead® Viability/cytotoxicity kit for mammalian cells, Molecular Probes™, ThermoFisher Scientific, Inc., Waltham, MA). In short, at least 50 μl dye solution (2 μM Calcein AM and 4 μM Ethidium homodimer‐1 [EthD‐1]) was added to scaffolds and incubated at RT in the dark for 30 min. Scaffolds were imaged at ×100 magnification with extended focus using an automated Axio Imager.Z1 (Zeiss) with excitation and emission wavelengths of either 488 and 517 nm (Alexa Fluor) for Calcein AM or 528 and 617 nm (Propidium Iodide) for EthD‐1, respectively. The ratio of live and dead cells was determined in triplicate with ImageJ.

### 2.4 Primary cell proliferation and differentiation

#### 2.4.1 *Culture conditions of rBMSC*

To identify differences in proliferation and differentiation between TFM types, primary rBMSCs isolated from femurs of a 9 week old male Wistar rat (local approval number RU‐DEC 2014007, Radboudumc Nijmegen, The Netherlands) were seeded onto TFM scaffolds according to standard protocol (Walboomers, Elder, Bumgardner, & Jansen, 2006). Rat femurs were carefully excised, wiped down, and thoroughly washed three times in washing medium (α‐MEM supplemented with 0.5 mg/ml gentamicin (Gibco) and 2.5 μg/ml fungizone (Amphotericin B, Gibco). The epiphyses were cut off, and contents of the diaphyses were flushed out with α‐MEM. The cell suspension was thoroughly resuspended, divided over three 75 cm^3^ culturing flasks, and pre‐cultured in complete medium at 37°C and 5% CO_2_ for 7 days to allow for proliferation. On Day 2, medium was changed to remove dead and non‐adherent cells. Following proliferation, cells were enzymatically dissociated from culture flasks, taken up in 9 ml complete medium, centrifuged at 250 rcf for 5 min and resuspended in differentiation medium (complete medium supplemented with 10 nM dexamethasone, 28 mM l‐ascorbic acid and 10 mM β‐glycerolphosphate [all: Sigma]). A total of 1.5 × 10^6^ cells were seeded onto the TFM scaffolds relative to the TFM volume (Table 2), using the method described in Section 2.3. An equal seeding density was used for all TFM types to ensure fair comparison of results. Following seeding, scaffolds were transferred to individual wells of regular 24‐wells plates containing 1 ml differentiation medium, and cultured for 3, 7, 14, or 21 days. Medium was refreshed twice a week.

**Table 2:** The number of cells seeded onto TFM scaffolds

TFM type	Cells/scaffold ×10^3^	Seeding density (×10^3^ cells/mm^3^)
22–60	6.3	1.5
22–70	6.3	1.5
22–80	6.3	1.5
35–60	6.3	1.5
35–70	6.3	1.5
35–80	6.3	1.5
50–70	9.4	1.5
50–75	9.4	1.5
50–87	18.8	1.5

Abbreviation: TFM, titanium fiber mesh.

#### 2.4.2 *SEM*

After 3, 7, 14, and 21 days of culture, samples were transferred to new 24‐wells plates, washed in PBS and fixed in 2% glutaraldehyde (Merck KGaA, Darmstadt, Germany) in 100 mM cacodylic acid buffer (pH 7.4, Serva Electrophoresis GmbH, Heidelberg, Germany) for 10 min. Samples were rinsed with PBS and dehydrated in a graded series of ethanol (70–100%) before drying in tetramethylsilane (TMS, Sigma‐Aldrich Chemie GmbH, Steinheim, Germany). Afterward samples were coated with 10 nm chromium, and SEM imaging was done as described in Section 2.1.1.

#### 2.4.3 *DNA quantification*

DNA quantifications were performed after 3, 7, 14, or 21 days of culture. Scaffolds were washed twice with PBS, transferred to 2 ml Eppendorf tubes containing 1 ml MilliQ, and stored at −20°C. Upon analysis, cells were lysed with three cycles of freeze‐thawing, after which the scaffolds were sonicated for 10 min, resuspended and centrifuged for 5 min at 10,000 rcf to eliminate large cell fragments. DNA quantifications were performed on the supernatant with a QuantiFluor® dsDNA System kit (Promega Corporation, Madison, WI) according to manufacturer's protocol. In short, 100 μl sample was added to 100 μl DNA dye reagent in clear 96‐wells plates and incubated at RT in the dark for 5 min. Fluorescence intensity was measured at excitation and emission wavelengths of 490 and 535 nm, respectively. Λ phage dsDNA was used to prepare a standard curve.

#### 2.4.4 *Alkaline phosphatase assay*

Expression of alkaline phosphatase (ALP) was measured using the supernatant prepared for DNA quantification. In triplicate, 80 μl sample was added to 20 μl 0.5 M 2‐amino‐2‐methylpropanol buffer solution (Sigma) in a 96‐wells plate. Upon addition of 100 μl of 5 mM ALP substrate p‐nitrophenyl phosphate (PNP, Sigma), plates were incubated at 37°C for 1 hr, and 100 μl 3 M NaOH (Merck) was added to stop the reaction. Turnover of PNP to 4‐nitrophenol was determined by measuring absorbance at a wavelength of 405 nm, and normalized to the amount of DNA.

#### 2.4.5 *Quantification of calcium in extracellular matrix*

Scaffolds were transferred to new 2 ml Eppendorf tubes containing 1 ml 0.5 M acetic acid (Scharlau, Scharlab S.L., Sentmenat, Spain), left at RT overnight, and resuspended. Ten microliters of sample was added to 300 μl working reagent (o‐cresolphtalein complexone (50 μg/ml, Sigma), 8‐hydroxyquinoline (1 mg/ml, Sigma), ethanolamine‐boric acid buffer (14.8 M, pH 11, Merck and Scharlau), and MilliQ in a 5:2:5:88 ratio) in 96‐wells plates. Plates were incubated for 5 min at RT and calcium concentration was measured at 560 nm. CaCl_2_ was used to prepare a standard curve.

### 2.5 Statistical analyses

Data are presented as mean with SD. Data for DMA and seeding efficiency were analyzed using One‐Way ANOVA's with Tukey's multiple comparison test. Data for DNA, ALP activity, and Ca quantifications were analyzed using Two‐Way ANOVA's with Bonferroni's posttest. Results were considered statistically significant when *p* values lower than .05 (*p* < .05). All statistical analyses were performed with Graphpad Prism 5 (Graphpad Software, Inc., San Diego, CA).

## 3 RESULTS

### 3.1 TFM characterization

Characteristics of nine types of TFM are summarized in Table 3. The TFM sheets varied in fiber size and porosity were obtained and studied for area density, porosity (theoretical and total porosity), pore sizes, fiber dimensions and stiffness. Porosity, pore sizes, and fiber dimensions were measured from SEM images of the cross‐section.

**Table 3:** Overview of characteristics of TFM

		22–60	22–70	22–80	35–60	35–70	35–80	50–70	50–75	50–87
Area density	(g/m^2^)	603	432	305	540	390	225	500	510	600
Theoretical porosity	(%)	56	68	78	60	71	83	78	77	87
Total porosity	(%)	36.8 ± 1.4	54.9 ± 6.4	72.9 ± 1.0	33.3 ± 2.6	58.3 ± 2.5	83.7 ± 5.6	52.1 ± 8.5	64.1 ± 3.6	80.0 ± 0.7
Pore size	(μm)	25.83 ± 9.23	42.68 ± 14.40	71.90 ± 19.16	61.71 ± 16.95	73.06 ± 19.27	133.28 ± 30.85	84.85 ± 26.79	102.29 ± 25.15	159.20 ± 28.17
Fiber dimensions: Length	(μm)	23 ± 5	17 ± 2	21 ± 3	43 ± 6	47 ± 7	42 ± 15	60 ± 4	63 ± 5	46 ± 6
Fiber dimensions: Width	(μm)	8 ± 5	7 ± 2	10 ± 3	18 ± 2	19 ± 7	23 ± 4	22 ± 5	20 ± 10	30 ± 5
Stiffness at 1 μm and 1 Hz	(GPa)	33.60 ± 5.46	26.95 ± 8.89	19.83 ± 12.0	46.41 ± 8.67	32.23 ± 3.21	33.25 ± 1.77	7.58 ± 2.44	9.30 ± 1.70	4.30 ± 0.73

Abbreviation: TFM, titanium fiber mesh.

#### 3.1.1 *TFM morphology*

**Figure 1:**
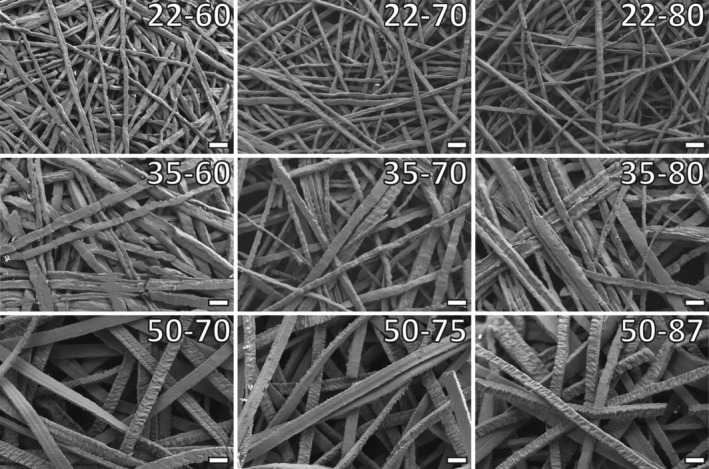
Overview of SEM images of TFM sheet surfaces. Images were made at ×250 magnification. Scale bars represent 100 μm. The TFM types with 22 μm fibers appear almost molten and show a smooth fiber surface. Fibers are densely packed. Types with 35 μm fibers show this molten look as well. Note the presence of fibers that have fused, most clearly seen in TFM type 35–80. Fibers of TFM types 50–70 and 50–75 appear flattened, with both smooth and rough sides. TFM type 50–87 shows these rough and smooth sides as well, but fibers are more cubic. TFM, titanium fiber mesh

SEM images of the surface of the TFM sheets revealed randomly oriented interwoven fibers for all nine mesh types (Figure 1). The fibers in all TFM types had a rectangular shape, but this shape was more pronounced for the 50 μm fibers. Also, for all fiber sizes, one side of the fiber had a rough surface, while the other side showed a smooth appearance. Again, this was more pronounced for the 50 μm fibers. Further, in the TFM 35 type, fusion of titanium fibers was observed. SEM pictures demonstrated that mesh porosity decreased with the reduction of the fiber size, that is, porosity decreased as follows TFM22 < TFM35 < TFM50. Finally, SEM‐EDS confirmed that all materials were pure titanium without other compositions (Supporting Information).

#### 3.1.2 *TFM porosity*

**Figure 2:**
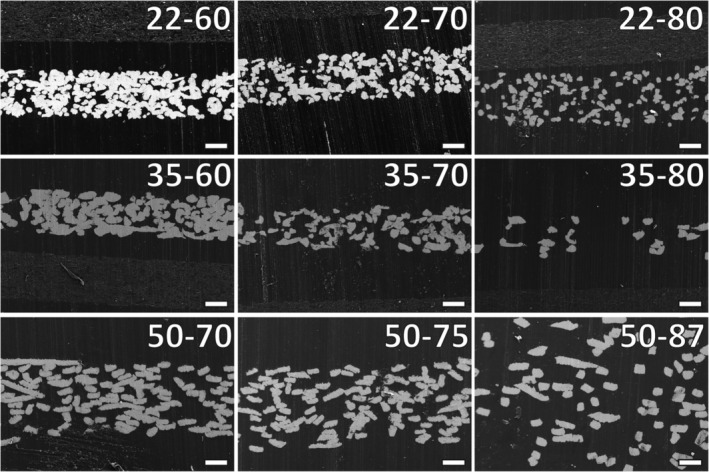
Porosity of TFM. TFM was embedded in methylmethacrylate (MMA). Cross‐sections were made and were visualized with SEM imaging at ×200 magnification. Scale bars represent 100 μm. Note that the thickness of the sheets is clearly visible, with type 50–87 even exceeding the image boundaries. TFM, titanium fiber mesh

The porosity of the TFM was measured with SEM (Figure 2). Cross‐sections showed a clear contrast between the electron‐conducting fibers and surrounding MMA. Note the differences in the thickness of the several types of TFM sheets. As is shown in Table 3, total porosity measured from SEM images was not always according porosity as specified by the manufacturer; whereas the theoretical porosity calculated from the (area) density was much more similar.

#### 3.1.3 *TFM pore size*

**Figure 3:**
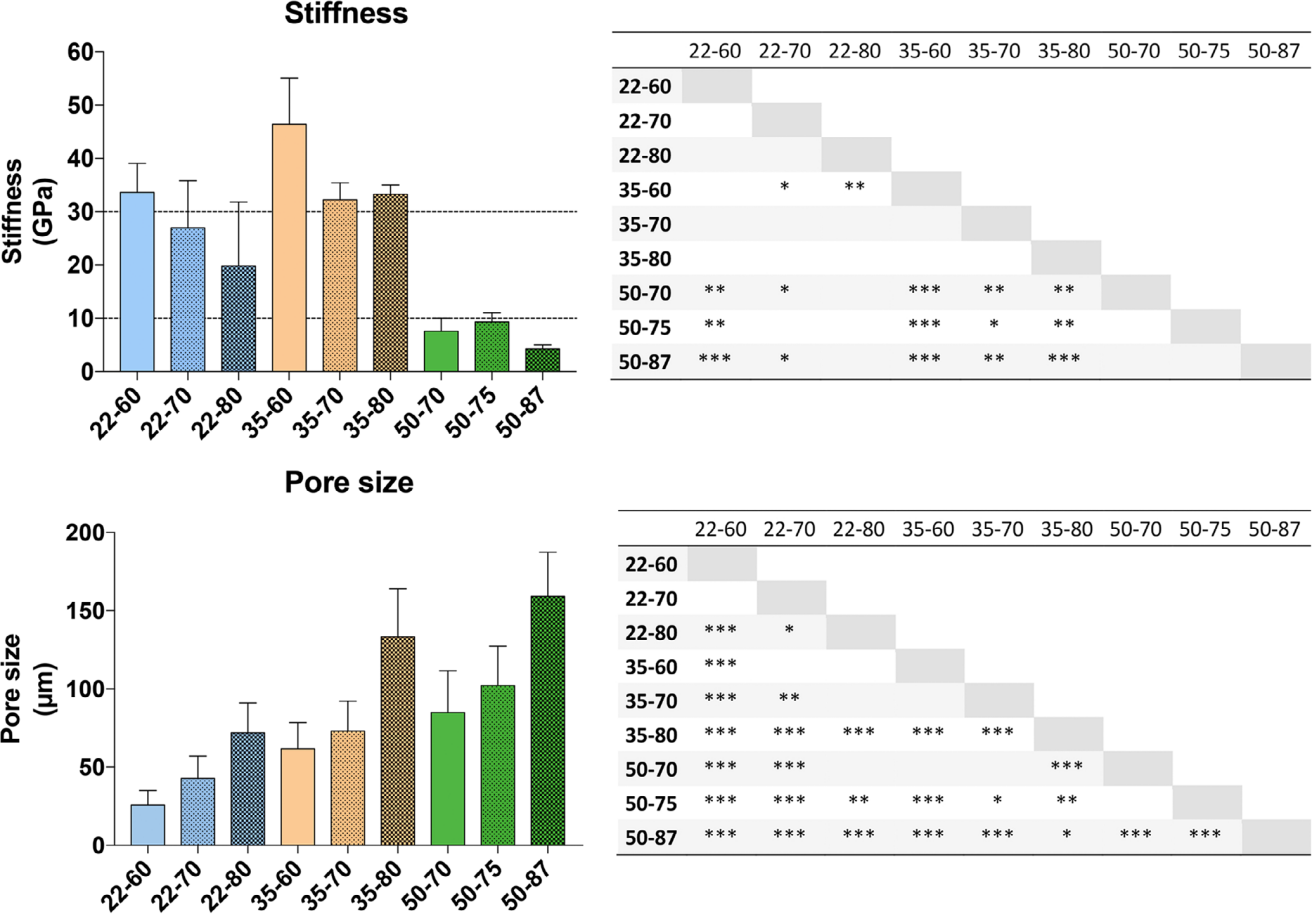
Flexibility of TFM as measured with stiffness and pore size as measured with the cross‐sections. Stress was applied to TFM strips with dynamic mechanical analysis (DMA), with an amplitude of 1 μm and a frequency of 1 Hz. The pore size was also determined via cross‐sections. Up left: Stiffness of the TFM types. Dotted lines indicate a range of Young's moduli for cortical and trabecular bone samples of 2 mm thickness. Down left: pore sizes of the TFM types. Right: significant differences between TFM types: *p < .05; **p < .01; ***p < .001. Apart from TFM type 22–80, the TFM types with 50 μm fibers have significantly lower stiffness than all other types (thus are more flexible). TFM type 35–60 had a significantly higher stiffness than all other types, except type 22–60. TFM type 50–87 had a significantly higher pore size (~150 μm) than all other types. TFM, titanium fiber mesh

The distance between fibers in each TFM samples was also measured in the SEM cross‐sections (Figure 3). TFM 50–87 had the largest pore size of 159.20 ± 28.17 μm, while TFM 22–60 had the smallest pore size of 25.83 ± 9.23 μm. For the meshes of the same fiber size, the pore size increased along with the volumetric porosity. Besides, for the TFM types with the same volumetric porosity, the sample with thicker fiber size had a larger pore size.

#### 3.1.4 *TFM flexibility*

The stiffness, an indicator of the flexibility of TFM, was measured with DMA at a standardized amplitude of 1 μm and a frequency of 1 Hz (Figure 3). TFM types with 50 μm fibers had significantly lower stiffness than most other types, whereas the stiffness of type 35–60 was significantly higher. No significant differences were found within groups of TFM scaffolds with 22 or 50 μm fiber size. Evidently, the stiffness for TFM are lower than the stiffness for solid cpTi (105 GPa) (Abdel‐Hady Gepreel & Niinomi, 2013). As a reference, a range of Young's moduli (10–30 GPa) for cortical and trabecular bone of 2 mm thickness is included in the graph (Rho, Tsui, & Pharr, 1997).

### 3.2 Proliferation on TFM scaffolds

#### 3.2.1 *Cell viability*

**Figure 4:**
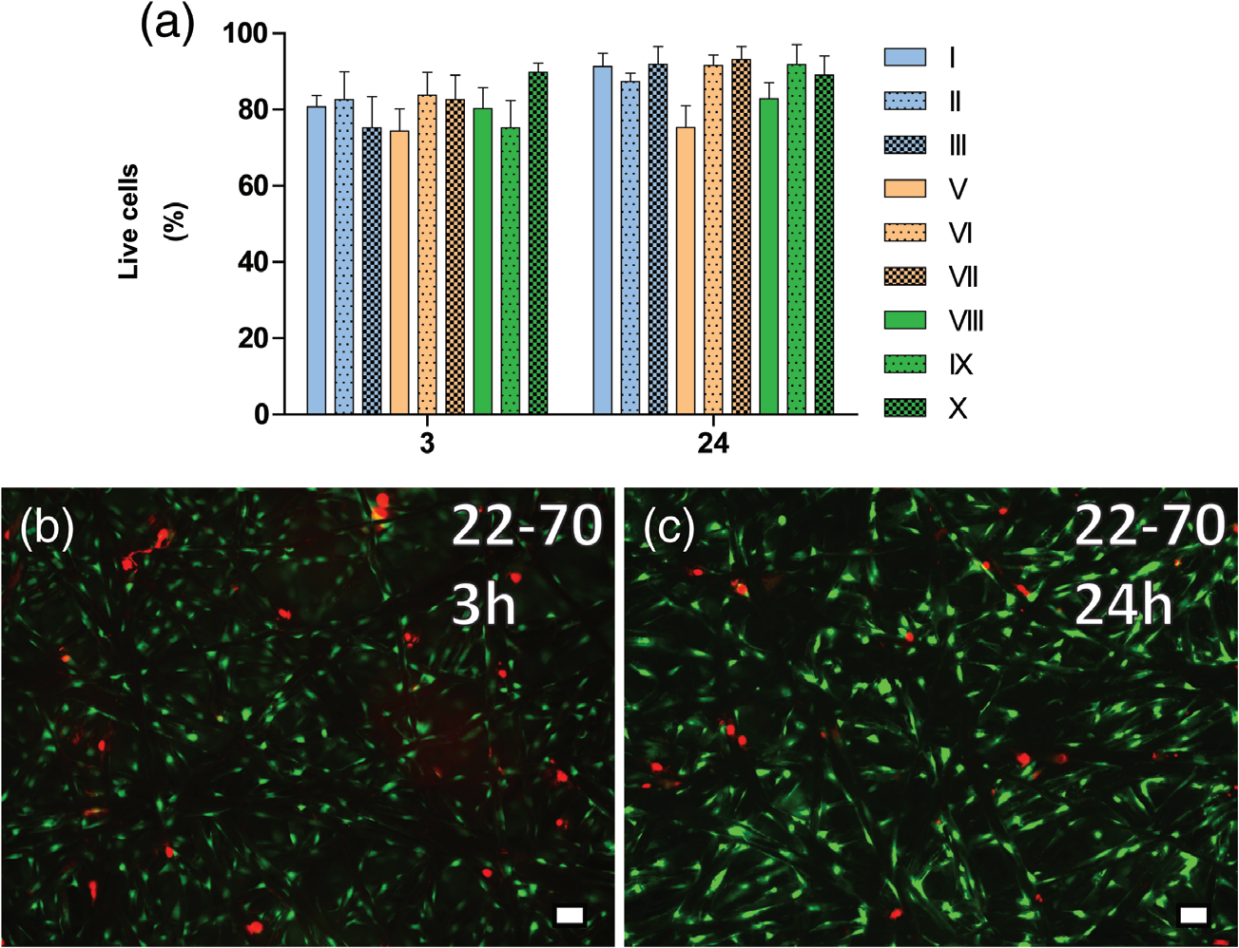
Cell viability after dynamic seeding on TFM. MC3T3 cells were dynamically seeded for 3 hr under rotation, at 2 Hz and tilted at 50°. Cell viability was measured with Live/Dead staining of cells. (a) Cell viability for all TFM types after 3 and 24 hr of seeding/culture. Cell viability was found adequate for ensuing experiments. (b + c) Typical image of Live/Dead staining after 3 or 24 hr, at ×100 magnification. Scale bar represents 50 μm. Green, viable cells; red, dead cells. TFM, titanium fiber mesh

Cell viability was assessed with a Live/Dead staining of MC3T3 cells after 3 hr of dynamic seeding onto TFM scaffolds and after 21 hr of incubation (i.e., total of 24 hr, Figure 4). Images showed an abundance of viable cells (green) and a few dead cells (red) that had adhered to the TFM fibers. Quantification of red and green cells revealed that cell viability ranged from 74.4% ± 5.8% to 89.8% ± 2.4% or from 75.3% ± 5.7% to 93.1% ± 3.4% after 3 or 24 hr, respectively.

#### 3.2.2 *Proliferation, qualitative*

**Figure 5:**
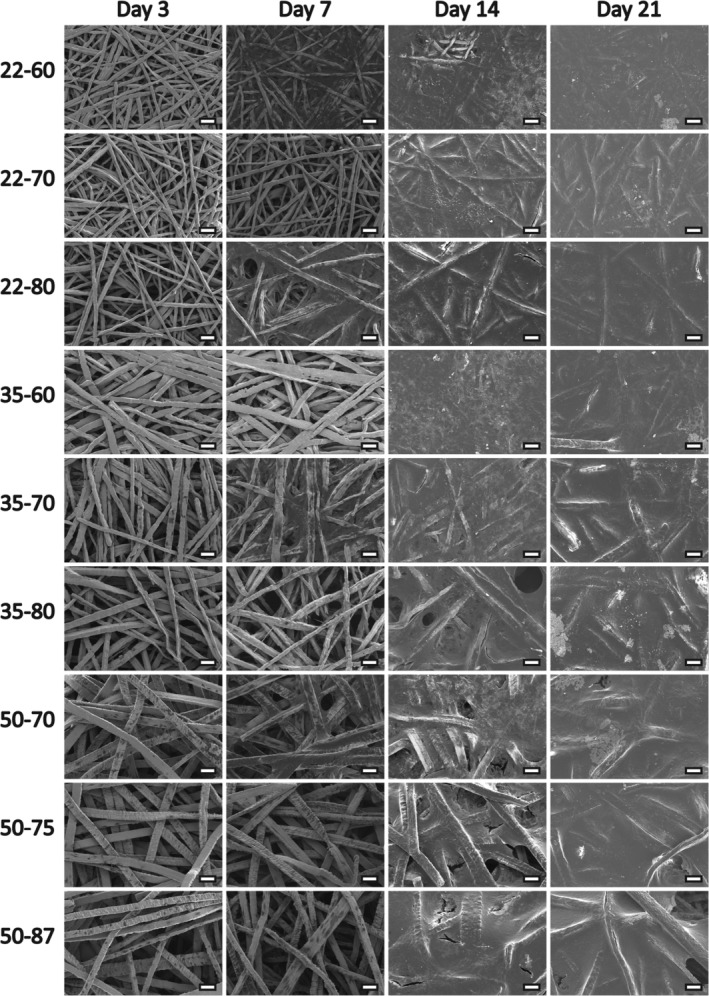
Overview of proliferation of rBMSCs on all types of TFM. After 3, 7, 14, and 21 days of culture, scaffolds were visualized with SEM imaging at ×250 magnification and 10 kV. Scale bars represent 100 μm. Over time, cells proliferate and form a layer over the TFM surface. rNMSCs, rat bone marrow‐derived stromal cells; TFM, titanium fiber mesh

**Figure 6:**
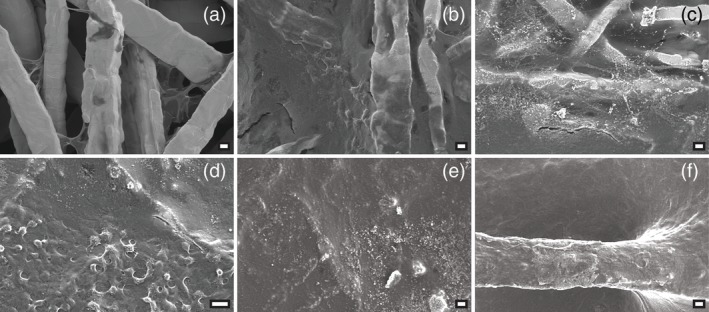
Details during proliferation of rBMSCs on TFM. SEM images were made at 1000x magnification at 10 kV. Scale bars represent 10 μm. (a) TFM type 35–70 at Day 3. Cells bridge the gaps between the fibers, or appear as dark spots on the fibers. (b) TFM type 35–70 at Day 7. Cells grow over the fiber surface. (c) TFM type 22–60 at Day 14. Depositions of mineralization appear. (d) TFM type 22–70 at Day 14. Cells appear rounder, characteristic of osteoblasts. (e) TFM type 22–70 at Day 21. Depositions of mineralization on a closed layer of cells. (f) TFM type 35–60 at Day 21. A layer of cells covers an extruding fiber. rNMSCs, rat bone marrow‐derived stromal cells; TFM, titanium fiber mesh

Scaffolds of all nine TFM types were seeded with rBMSCs and cultured for 3, 7, 14, or 21 days. SEM images at Days 3, 7, 14, and 21 showed the formation of a layer composed of both cells and some extracellular matrix covering the surface (Figure 5). At Day 3, cells were found to be stretched out between fibers and bridged the mesh pores (Figure 6a). At Day 7, cells started to form a layer that covered the fiber surface and mesh porosity (Figure 6b). From Day 14 on, a multilayer of cells was observed over the surface of the scaffolds. Some cells had a rounded morphology, which indicates osteoblastic differentiation (Figure 6d). At Day 14, small globular calcium phosphate mineral depositions were seen (Figure 6c,e) (Matsuzaka, Walboomers, Yoshinari, Inoue, & Jansen, 2003). At locations, where fibers protruded out of the TFM, a clear view was obtained of the guidance of the cells over the mesh fibers and into the mesh porosity (Figure 6f).

#### 3.2.3 *Proliferation, quantitative*

**Figure 7:**
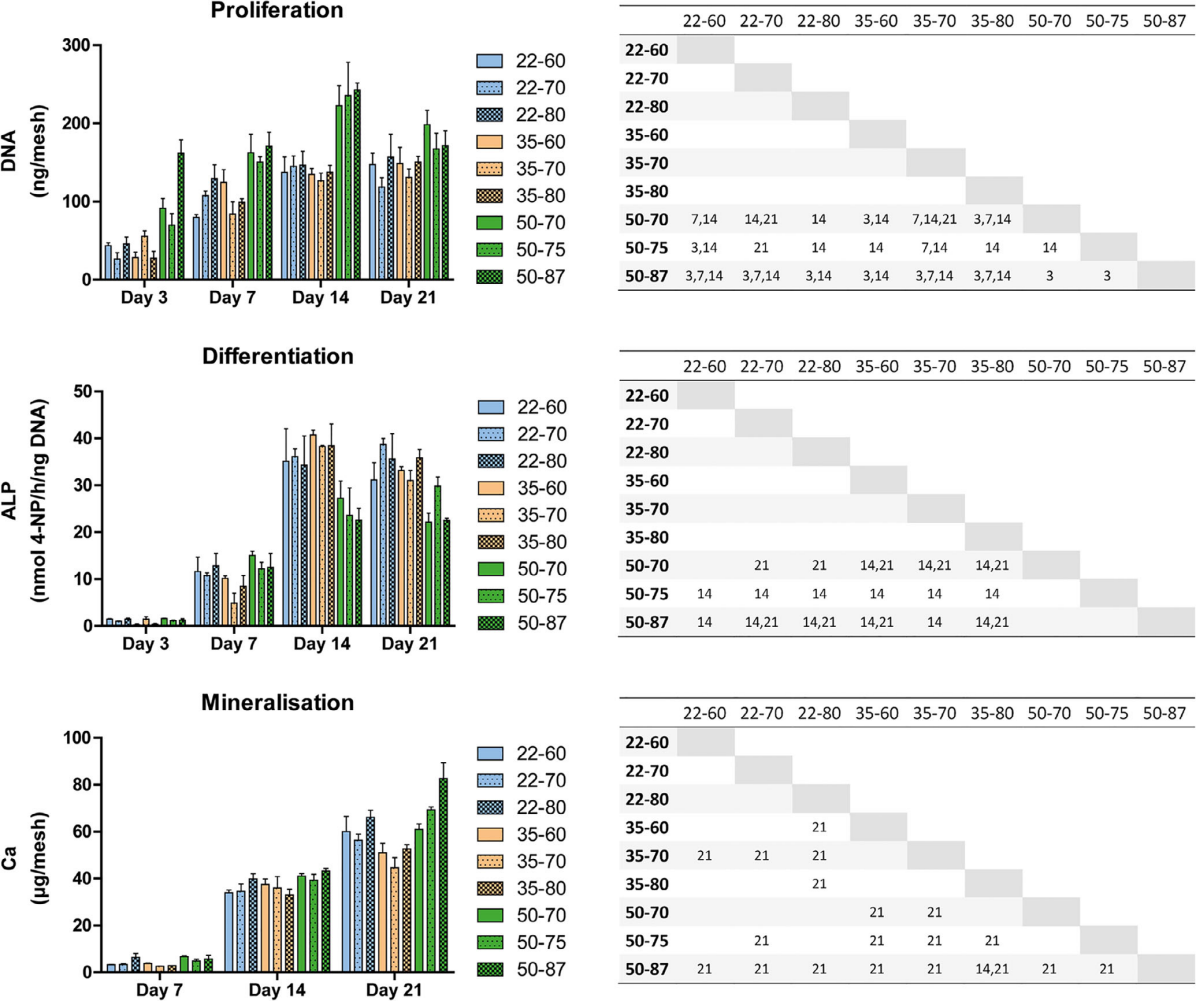
Behavior of rBMSCs during culture on TFM. TFM was cultured for 3, 7, 14, or 21 days. DNA quantity, alkaline phosphatase (ALP) activity and calcium deposition were measured in triplicate. Due to the large amount of data in each graph, significant differences are shown in tables. The numbers indicate the time point at which a combination of TFM types showed a significant difference: *p* < .05. *Proliferation*: Proliferation measured by DNA quantification. TFM types with 50 μm fibers often contained significantly more DNA compared to other types, mainly at Day 14. *Differentiation*: Differentiation measured by ALP activity. TFM types with 50 μm fibers often showed significantly lower ALP activity, mainly at Days 14 and 21. *Mineralization*: Mineralization measured with calcium quantification. Calcium contents showed significant differences only at day 21. TFM type 35–70 remained significantly lower in calcium, whereas TFM type 50–87 was significantly higher than all other types. TFM, titanium fiber mesh

Total DNA quantifications were performed after 3, 7, 14, and 21 days of culture. The amount of DNA increased in all TFM types over time (Figure 7). Particularly with regard to TFM 50 types, the amount of DNA decreased at Day 21. For TFM 50 types, amount of DNA was significantly higher at Days 3, 7, and 14 compared with the TFM 22 and 35 type, but at Day 21 DNA amount of TFM 50 decreased.

### 3.3 Osteogenic differentiation on TFM scaffolds

#### 3.3.1 *ALP activity*

ALP activity was used as a marker for osteoblastic differentiation. Figure 7 shows the characteristic increase in ALP activity over time for all TFM types, with the peak of activity at Day 14. Specifically, at Day 14, ALP activity of TFM with 50 μm fibers was significantly lower than ALP activity of TFM with 22 or 35 μm fibers. There were no significant differences between 22 and 35 μm TFM's at any time point.

### 3.4 Mineralization on TFM scaffolds

As an evaluation of the capacity to form a mineralized extracellular matrix, the amount of calcium on TFM samples was measured. All TFM types contained increasing quantities of Ca (Figure 7), indicating that the cells were actively depositing mineralized matrix over time. Spontaneous mineralization did not occur, as TFM incubated without cells in the same media and at the same frequent medium change, did not show Ca deposition (data not shown). At Day 21, calcium deposition on TFM 50–87 was significantly higher compared to all other TFM types. Mineralization on TFM 35–70 was significantly less pronounced compared to TFM with 22 or 50 μm fibers.

## 4 DISCUSSION

The aim of this study was to evaluate the stiffness and in vitro cell response to TFM scaffolds with different network architecture. Nine constitutions of TFM with unique fiber thickness (22, 35, and 50 μm) and porosity (60, 70, 75, 80, 87%) were examined. The results indeed confirmed that the tailoring of fiber size and porosity was an effective means to reach stiffness from 4.3 ± 0.73 GPa to 46.41 ± 8.67 GPa. Also, TFM scaffolds of all types were able to support cell growth and differentiation, as shown by SEM imaging and by the increase in DNA contents, ALP activity, and matrix mineralization over time.

To characterize the TFM types, the volumetric porosity and the pore size were validated on cross‐sections of TFM as described above. Compared to porous scaffolds with regular pore shapes and inerratic structures, TFM has the connective space in between the intricately distributed fibers, providing considerable space for cell migration and tissue ingrowth, but also make it difficult to accurately assess the pore distribution. Mercury intrusion porosimetry (MIP) and microcomputed tomography (μCT) are both widely used techniques for characterizing the distribution of porosity for scaffold materials (Lodoso‐Torrecilla et al., 2018). Unfortunately, they cannot provide convincing results for TFM. MIP has limitations when applied to materials that have irregular pore geometry (Abell, Willis, & Lange, 1999), and for TFM types with over 80% porosity, the flow of the mercury would occur without additional pressure, which results in the inaccuracy of the MIP analysis. Micro‐CT, as a nondestructive characterization technique, is also not applicable, due to the scattering arising from the titanium (Moore et al., 2004). Therefore, cross‐sections of the different TFM types were made to measure their porosity and pore size. The measured porosity (total porosity) was clearly lower than the porosity, as specified by the manufacturer. This discrepancy can be explained by our measuring method. Cross‐sections are subjected to the complications of longitudinally cut fibers: not all fibers are cut perfectly perpendicularly, which results in an overestimation of fiber area. For density measurement of porous materials, Archimedes' principle is a reliable method. However, because the narrow pore shape and distribution among the fibers of the TFM may create some amount of closed porosity, not accessible by fluids, Archimedes' principle may not be completely accurate. Therefore, the porosity was also calculated based on the density of the TFM sheets and was found similar to the specified porosity.

Porosity, as well as the pore size, has a strong influence on the mechanical properties of biomaterials; for instance, higher porosity always leads to lower stiffness (Karageorgiou & Kaplan, 2005). TFM types with 50 μm fibers had significantly lower stiffness than types with smaller fibers. In theory, if the porosity stays the same, a reduction in fiber size would increase the number of fibers: lumen and fibers would then be more finely distributed inside the TFM. This increase in the number of fibers will lead to a stiffer TFM with an increased stiffness, as was indeed the case for TFM types with fibers of 22 or 35 μm. Independent of these individual differences, all types of TFM show a markedly decreased stiffness compared to solid titanium (~105 GPa) (Abdel‐Hady Gepreel & Niinomi, 2013). For the 22 and 35 size fibers with the highest porosity, the stiffness approximate the values found in the literature for cortical bone (~30 GPa for samples of 2 mm thickness) (Rho et al., 1997). Of course, fiber orientation also has an effect on stiffness. Although the fibers we obtained in random directions, we cannot totally exclude the directional effects of the subsequent compaction and sintering process. Previously, other attempts have been made to lower stiffness of solid titanium implants by incorporating other metals (vanadium, aluminum, cobalt, molybdenum, or niobium). These methods can bring Young's modulus down to 55 GPa, yet it remains much higher than cortical bone (Abdel‐Hady Gepreel & Niinomi, 2013). Additionally, the incorporation of other metals gives rise to unfavorable changes in biocompatibility and resistance against corrosion, fatigue, and wear (Long & Rack, 1998). The use of TFM would thus be a better option. According to the diversity of stiffness, different types of TFM might be favorable for clinical use, or individual types of bone. TFM alone maybe not durable as a load‐bearing implant due to its strength, but still previous studies have already indicated that a so‐called mesh porous coated system used on orthopedic devices was indeed robust enough for clinical application (Jasty, Bragdon, Haire, & Harris, 1993; Stilling et al., 2011). Admittedly, the application of TFM used as a surface layer on orthopedic and dental implants has the potential risk that coated implants fail primarily from the layers shearing off the implant. Therefore, follow up investigation on optimization for implant and TFM design is required in preclinical implantation studies.

The flexible porous structure should be manufactured by balancing pore size and porosity to maintain mechanical properties, that is, stiffness, as well as achieve appropriate bone ingrowth. The pore size of TFM will decrease when the fiber size is reduced and volumetric porosity is maintained simultaneously or of the volumetric porosity is reduced and fiber size is maintained simultaneously (Figure 3). Still, the pore sizes from all TFM types correspond to pore sizes that are generally considered optimal to support the growth of bone forming cells (50–500 μm) (Tadic, Beckmann, Donath, & Epple, 2004). A constant increase in the number of cells was seen in all samples quantified by DNA and confirmed by SEM images. Notably, TFM with 50 μm fibers showed at Days 3, 7, and 21 significantly higher DNA contents compared to other TFM types. It can be assumed that a higher fiber size results in increased cellular adhesion, which results in an enhanced cellular growth. However, it was also observed that at Day 3, DNA content at TFM 50–87 was significantly higher than DNA content at TFM 50–75 and 50–70. This suggests that although there can be an initial boost in cell numbers, this does not cause the higher DNA amounts at later time points. Thus, a better explanation would be the effect of larger pore size (from 84.85 ± 26.79 μm to 159.20 ± 28.17 μm) of TFM with 50 μm fiber, which facilitated transport of oxygen and nutrients (Takahashi & Tabata, 2004). Further, a decrease in DNA amount on TFM 50‐types occurred at Day 21. This effect was seen in earlier studies with TFM and is due to the increased mineralization, which does not allow a complete retrieval of all DNA out of the specimens (van den Dolder, Bancroft, et al., 2003a)

Differentiation was studied by quantifying the ALP as an early marker for osteogenicity. Although more cells adhered to the TFM types with 50 μm fibers, after correction for cell number these fibers generally showed least ALP activity. The decrease in ALP activity can be explained by the promoted proliferation and also the bigger pore size in TFM types with 50 μm fibers. The combination of relatively high porosities (87, 70, and 75%) with bigger fibers will result in larger spaces in between the fibers, which leads to more distance between individual cells and thus less cell–cell contact. Cell–cell interaction has proven to play an important role in osteodifferentiation: bone marrow‐derived stem cells express a range of cadherins (integral transmembrane proteins that facilitate cell–cell adhesion and interaction), that change upon differentiation to the osteogenic lineage (Stains & Civitelli, 2005). In vitro, disruption of cadherin functioning impaired the osteoblastic differentiation (Cheng, Shin, Towler, & Civitelli, 2000; Ferrari et al., 2000), and in mice, the transgenic expression of a dominant negative N‐cadherin mutant (which impedes cell–cell adhesion normally facilitated by cadherins) caused the peak bone mass to occur later in life, suggesting problems in osteogenic differentiation (Castro et al., 2004). Therefore, decreasing cell–cell contact may decrease cadherin‐mediated interactions, and in turn delay osteoblastic differentiation.

Differentiation was also studied by quantifying matrix mineralization, assessed by Ca deposition of cells. All samples showed evident matrix mineralization. Although individual differences exist, these seem rather small and are deemed not relevant. It seemed that 50 and 22 fibers exhibited more mineralization than 35 fibers, indicating that appropriate pore size and porosity are required for homogenous cell distribution, fluid flow, and diffusion of nutrients and oxygen, and thus may impact the way rBMSCs deposit mineralization (Bencherif, Braschler, & Renaud, 2013). In addition, cellular behavior is undoubtedly affected by the applied strain (Diederichs, Freiberger, & van Griensven, 2009), which would be influenced by different stiffness of diverse TFM types under loading condition. Further research should be undertaken to investigate the stiffness and loading combined effect on the cellular behavior on meshes. It should be noted that the minimum porosity or pore size necessary for osteogenesis in vivo will be different and that in vivo osteogenesis is also depending on other processes, for example, vascularization (Karageorgiou & Kaplan, 2005). This underlines again that follow up investigation in preclinical implantation studies is required to make definitive conclusions.

Before concluding, it should be noted that some confounding effects can never be excluded. Alterations to the TFM in terms of fiber dimensions and porosity will inevitably impact other morphological parameters. SEM images showed that besides fiber size and porosity, the surface roughness and density of the TFM varied between types, which should be taken in consideration when assigning the results to specific parameters such as fiber size or porosity. As shown in SEM images, TFM types with 35 μm fibers showed more fusion of fibers than other TFM types, and TFM types with 50 μm fibers displayed rough sides, whereas the other types appeared somewhat smoother. These effects are the consequence of different settings (e.g., sintering) during the manufacturing process of the various TFM types. It is already known that cells react to such micro‐ and nanoscale structures. Increasing roughness (e.g., by sandblasting, acid etching, and nanotube formation) of titanium surfaces is known to result in increased initial adhesion of cells (Dhaliwal et al., 2017; Huang et al., 2017; Mustafa et al., 2001)

## 5 CONCLUSIONS

The current study evaluated stiffness and in vitro cell response to TFM scaffolds with different network architecture. An overview of characteristics of each TFM type was provided so that choices can be made on their inclusion in future studies, based on the specific desired application. Changes in stiffness could effectively be achieved by tailoring fiber size or porosity, which will alter the pore size simultaneously. Regarding cellular behavior, it has become clear that all TFM types support growth and differentiation of bone cells. Further preclinical (in vivo) studies are needed to assess which TFM types are most suitable as clinical use for bone ingrowth and bone regeneration.

## Supporting information


**Figure S1** SEM‐EDS images of TFM sheet surface.
