# Supplementary figures and images for: Porous titanium fiber mesh with tailored elasticity and its effect on stromal cells
Source: J Biomed Mater Res B Appl Biomater. 2020 Jan 14;108(5):2180–91. doi: 10.1002/jbm.b.34556 (PMC7217192; doi:10.1002/jbm.b.34556)

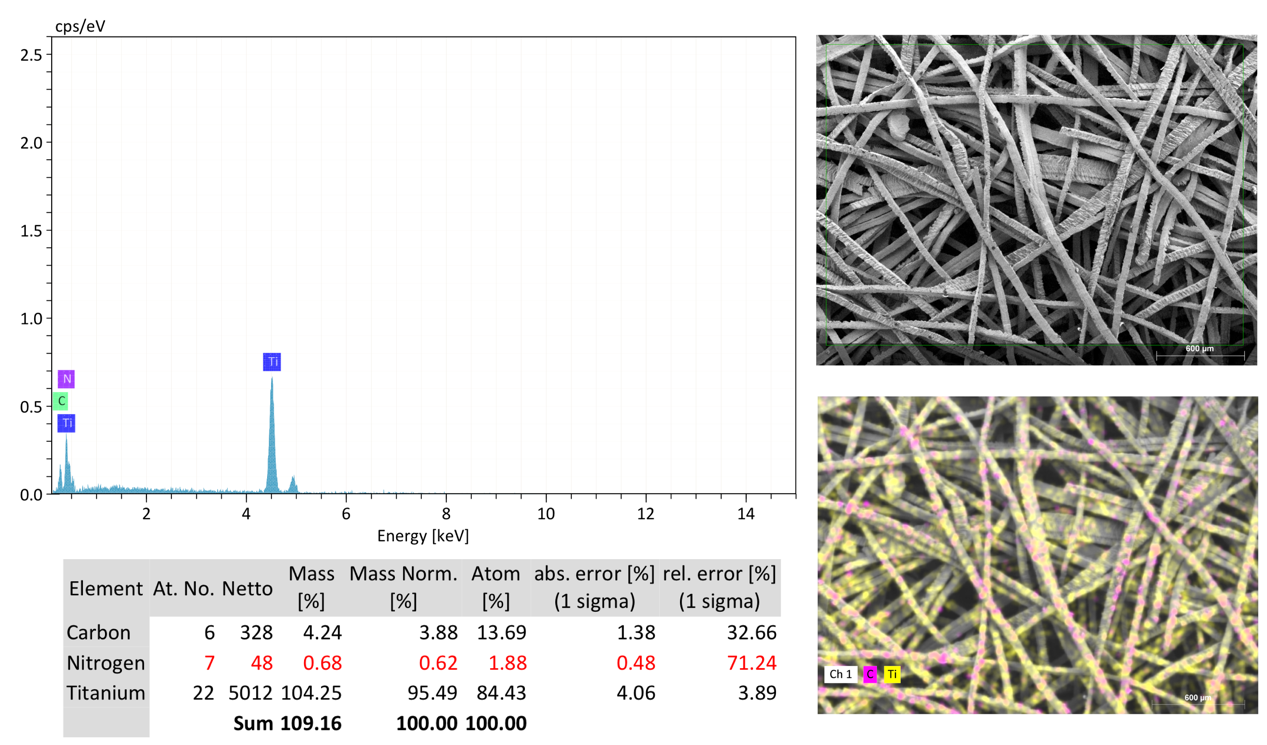


Figure S1. SEM-EDS images of TFM sheet surface.

Supplement: Supplementary file 1 — Figure S1 SEM‐EDS images of TFM sheet surface. [file JBM-108-2180-s001.docx]
